# Dynamic finite element modelling of the macaque mandible during a complete mastication gape cycle

**DOI:** 10.1098/rstb.2022.0549

**Published:** 2023-12-04

**Authors:** Olga Panagiotopoulou, Dale Robinson, Jose Iriarte-Diaz, David Ackland, Andrea B. Taylor, Callum F. Ross

**Affiliations:** ^1^ Monash Biomedicine Discovery Institute, Department of Anatomy and Developmental Biology, Monash University, Melbourne, Victoria 3800, Australia; ^2^ Department of Biomedical Engineering, University of Melbourne, Melbourne, Victoria 3053, Australia; ^3^ Department of Biology, University of the South, Sewanee, TN 37383, USA; ^4^ Department of Foundational Biomedical Sciences, Touro University California, Vallejo, CA 94592, USA; ^5^ Department of Organismal Biology and Anatomy, University of Chicago, Chicago, IL 60637, USA

**Keywords:** chewing, feeding, finite element analysis, diet, food mechanical properties, strain

## Abstract

Three-dimensional finite element models (FEMs) are powerful tools for studying the mechanical behaviour of the feeding system. Using validated, static FEMs we have previously shown that in rhesus macaques the largest food-related differences in strain magnitudes during unilateral postcanine chewing extend from the lingual symphysis to the endocondylar ridge of the balancing-side ramus. However, static FEMs only model a single time point during the gape cycle and probably do not fully capture the mechanical behaviour of the jaw during mastication. Bone strain patterns and moments applied to the mandible are known to vary during the gape cycle owing to variation in the activation peaks of the jaw-elevator muscles, suggesting that dynamic models are superior to static ones in studying feeding biomechanics. To test this hypothesis, we built dynamic FEMs of a complete gape cycle using muscle force data from *in vivo* experiments to elucidate the impact of relative timing of muscle force on mandible biomechanics. Results show that loading and strain regimes vary across the chewing cycle in subtly different ways for different foods, something which was not apparent in static FEMs. These results indicate that dynamic three-dimensional FEMs are more informative than static three-dimensional FEMs in capturing the mechanical behaviour of the jaw during feeding by reflecting the asymmetry in jaw-adductor muscle activations during a gape cycle.

This article is part of the theme issue ‘Food processing and nutritional assimilation in animals’.

## 1. Introduction

The finite element method is a powerful discretization approach of continuum mechanics problems posed by mathematically defined statements [1], first developed in the 1940s for use in structural engineering [1–4]. Over the last few decades, the finite element method has been extensively used to study the mechanical behaviour of biological systems and to test form-function hypotheses [5–27]. The processual component of the finite element method involves model creation, solution, post-processing and validation [28–30]. In brief, the geometry under consideration is first discretised into a number of elements connected at their vertices (nodes). For stress analysis, a variation in displacement (e.g. linear or quadratic) is assumed through each element, and equations describing the behaviour of each element are derived in terms of unknown nodal displacements. These equations are then combined to generate a set of system equations that describes the behaviour of the whole problem and are solved using the boundary condition [28–31].

Finite element models (FEMs) combined with rigid-body analysis have been used to study the function of the temporomandibular joint (TMJ) in humans [32,33], but to the best of our knowledge they have not been used to study mandibular strain, loading and deformation regimes during feeding. Instead, early research on the human and non-human primate mandible used three-dimensional static FEMs to study the mechanical behaviour of the feeding system and to test hypotheses on the role of food in species diversification and on adaptive specializations to environmental resources [5–10,34–37]. To determine how mastication on foods with different material properties affects deformation and strain regimes in the macaque mandible, we previously applied muscle force loading regimes recorded while the animal was chewing on three different food types (fresh grapes with skin, shelled nuts, dried fruits) to a series of subject specific, validated and static FEMs [5,6]. Our static FEMs modelled the 46th% of the gape cycle, which is the time when the highest bone strains were recorded *in vivo* by one of the strain gauges. The 46% of the gape cycle is close to the minimum gape, with gape angles at 0.4, 0.2 and 0.5° for dried fruit, grape and nuts chewing respectively (note: minimum gapes occur at 54% for dried fruit, 50% for grape and 60% for nut chewing). In addition, at 46% of the gape cycle the anterior temporales, working-side posterior temporales, superficial masseters and medial pterygoids have started to decline and the balancing-side deep masseters and posterior temporales are at their peak (electronic supplementary material, figure S1) [5]. This differential muscle activation results in dominant sagittal shear forces and sagittal bending moments in the balancing-side corpus; lateral transverse bending, and negative mediolateral (ML) twisting at the symphysis, and a combination of sagittal bending, anteroposterior twisting and lateral transverse bending moments in the working-side corpus [5]. At that time in the power stroke the areas of the mandible with the largest food-related variations in strain regimes extend from the lingual symphysis to the junction of the balancing-side corpus and ramus, and along the balancing-side medial prominence and endocondylar ridge. The medial prominence, torus triangularis and endocondylar ridge constitute the load path from the bite point to the balancing-side ramus and are probably important mandible features with which to infer function (diet) from form (shape) in macaques [5]. Similarities in feeding mechanics between chimpanzees, macaques and humans suggest that this may also be the case in living and fossil hominids [5,8,9].

Our FEM research on macaques [5] is, to our knowledge, the most detailed analysis of mandible feeding mechanics in a mammalian mandible yet published. However, to date this analysis has been static, only documenting strain, loading and deformation regimes at a single time point of the gape cycle, when peak bone strain magnitudes were recorded from a strain gauge at the inferior aspect of the lateral prominence of the working-side corpus [6]. Here we present the first dynamic FEM of a primate mandible during unilateral chewing, based on electromyography (EMG) data collected when the animal was chewing on three different food types: nuts, grapes and dried fruits. One goal of this analysis is to identify the times during the gape cycle at which the mandible experiences the highest loading and strain regimes. Although, the 46th% of the gape cycle is the time when the highest strains were recorded *in vivo*, it may not be the time point with the highest strains and moments in other areas of the mandible (away from the strain gauge location) during mastication. In addition, the 46th% of the gape cycle was not the point of maximum muscle EMG intensity, which is often used to load the FEMs. We asked whether the mandible is most highly strained at the same time during the gape cycle when chewing on different foods. A second goal is to examine dynamic changes in loading and strain regimes. The asymmetry in timing of the jaw-adductor muscles (and amplitude of activity) in primates during chewing suggest that a static FEM may not effectively represent the full complexity in mechanical behaviour of the jaw during feeding [38,39]. Because bone strain patterns and moments vary throughout the power stroke owing to variation in the activation peaks of the jaw-adductor muscles, we hypothesized that loading and strain regimes in the mandible also vary throughout the gape cycle, reflected in changing moments about the three anatomical axes. To address these goals, we built dynamic FEMs of a complete gape cycle using muscle force data from *in vivo* experiments to elucidate the impact of relative timing of muscle force on mandible biomechanics.

## 2. Methods

### (a) Overview

A detailed description of our methods is provided below. In brief, for the dynamic simulations we used two model variations: the ‘screws model’ and the ‘no-screws model’. The screws model is based on a previously validated static FEM of an adult rhesus macaque, which was constructed and loaded using *in vivo* data on muscle activations and three-dimensional mandible kinematics from the same animal [6]. For the measurement of the three-dimensional mandible kinematics, the animal had titanium screws implanted into its mandible and cranium. These screws created remodelling of the cortical and trabecular bone at the anterior mandible, however the impact of this bone remodelling on mandible mechanics is not known. To ensure that bone remodelling does not affect mandible mechanics during chewing for the scope of this study, we created a modification of the screws model, by removing all bone screws and the associated calluses virtually using Mimics v.25 and 3Matic v.17 software (Materialise, Belgium). We called this modified model, which has not been published before, the no-screws model. For transparency, we have included results from both model variations in this paper.

### (b) Model creation: model geometry, three-dimensional models and mesh files

As part of previous studies, the geometry of the skull was captured using computed tomography (CT) scans on a Philips Brilliance Big Bore scanner at the University of Chicago (isometric slice thickness 0.8 mm, 768 × 768 pixel images and 0.2 mm pixel size). Scans were processed in Mimics Materialise software v.17 to extract three-dimensional surface sets of the mandibular cortical bone, trabecular bone tissue, teeth, periodontal ligament and mandibular bone screws.

For finite modelling [5,6], three-dimensional surface data for the screws model were assembled in 3Matic v.10 (Materialise, Belgium) and converted into volumetric mesh files of linear tetrahedral elements (and hybrid for the periodontal ligament (PDL)), with maximum nominal size of 0.7 mm. For the no-screws model, we removed the screws and the associated bone remodelling virtually using Mimics v.25 and 3Matic v.17 software. During the screw removing and calluses process, we had to remove the PDL and assign the space it occupies to the cortical bone. The nominal element size and type for the no-screws model is similar to the screws model.

### (c) Model creation: material properties assignment

The cortical bone was modelled as orthotropic and heterogeneous using subject specific measurement of bone properties [6,40]. Linear elastic, isotropic and homogeneous properties were assigned to the trabecular bone tissue (*E* = 10 GPa; *v* = 0.3), and teeth (*E* = 24.5 GPa; *v* = 0.3) for both the screws and no-screws models and the bone screws (*E* = 105 GPa; *v* = 0.36) and periodontal ligament (*E* = 6.80 × 10**^−^**^4^ GPa; *v* = 0.49) for the screws model [6,13]. The material properties of the cortical bone were measured experimentally from the same animal using ultrasonic velocities [40] and theoretical modelling [6]. The material properties of the trabecular bone and the teeth are based on a previous sensitivity and validation analysis [6].

### (d) Model creation: loads and constrains

Both the screws and no-screws models were loaded and constrained in the exact same manner. All intersecting surfaces were bonded together using frictionless (tie) constraints. The simulated bite force of a complete gape cycle was modelled by constraining all translations at selected nodes on the occlusal surface of the left first premolar (P3), left second premolar (P4), and the left first molar (M1). The left (working) side mandibular condyle was fixed at one node against displacement in all directions and the right (balancing) condyle was fixed against superior-inferior (SI) and anterior-posterior (AP) displacement only [6].

Muscle force magnitudes were estimated using *in vivo* EMG data recorded when the animal fed on food items with different toughness and stiffness (soft food: grapes; dried fruit: prune, date, gummy bear^1^ [41], dried apricot/pineapple/cranberry; nuts: shelled almond, cashew, Brazil nut, walnut, pecan) [5,6] combined with subject specific muscle physiological cross-sectional areas (PCSAs) following the equation:

$$\begin{array}{rl} & \mathrm{PCSA}({\mathrm{cm}}^{2})=\frac{\left(\mathrm{muscle}\;\mathrm{mass}\;[g]\times \cos\theta \right)}{\left(\mathrm{fibre}\;\mathrm{length}\;[\mathrm{cm}]\times 1.0564\;g\;{\mathrm{cm}}^{-3}\right)}, \end{array}$$

where 1.0564 g cm^−3^ is the specific density of muscle [42], and fibre length is normalized using sarcomere length following protocols by Felder and colleagues [43–45].

Force estimates for the dynamic model were calculated as the mean normalized EMG magnitude during a complete gape cycle multiplied by the estimated PCSA multiplied by the specific tension of muscle (30 N cm^−2^) (see footnote^2^). Muscle forces were applied as amplitude functions at surface nodes representative of reference points of the insertions and origins of the working and balancing-side posterior and anterior temporales, superficial and deep masseters and medial pterygoids [6]. Muscle force vector orientations were calculated using the centroids of the origins on the cranium and the dynamically changing centroids of the insertions on the mandible. Electronic supplementary material, tables S1–S3 give the *x,y,z* components of the muscle force vectors assigned to the dynamic FEMs when the animal was chewing on nuts, dried fruit and soft food (i.e. grapes), respectively.

### (e) Model solution

The FEMs were solved in Abaqus CAE Simulia (v.2021) (Dassault Systémes, Vélicy-Villacoublay, France) using the Abaqus implicit solver at 100 increments of increment size of 0.0033 s as per the *in vivo* experiments.

### (f) Data post-processing

Axial, principal and shear strain regimes were visualized in figures representative of the complete gape cycle, with colours representing strain magnitudes. Loading regimes were quantified using moments. Specifically, moments were defined about the *Y* (AP axis) of the mandible and bending moments act about the orthogonal axes. Transverse bending moments were defined about the *X* (SI, vertical) axis, and sagittal bending moments act about the *Z* (transverse ML) axis. From Abaqus, we quantified moments about axes parallel to these coordinate axes but passing through centroids of cross sections of the hemimandibles and the symphysis.

## 3. Results

### (a) Dynamic changes in loading and deformation regimes across food types

#### (i) Loading regimes: moments

**Figure 1.**
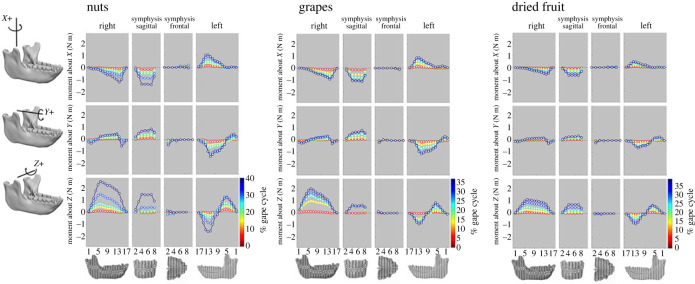
Moments (in N m; force (N) × distance (m)) acting about axes parallel to the coordinate system on the right (balancing) side, on the left (working) side, and through the symphyseal region of the macaque mandible at the no-screws model. Different line colours show moments acting on those sections at different times during the gape cycle; dark blue indicates moments at the time of peak moments. The precise percentage of the gape cycles for the three food types are: dry fruit, 5%, 14%, 22%, 31%, 39%; nuts, 5%, 14%, 23%, 31%, 40%; soft food, 5%, 13%, 21%, 29%, 35%. Numbers on the ordinate correspond to section planes illustrated in the figures at bottom. Moments about *X* (SI) axes are transverse bending moments; moments about *Y* (AP) axes are twisting moments; moments about *Z* (ML) axes are sagittal bending moments. Balancing-side frontal and working-side frontal: these moments are calculated as the sums of all the moments acting on the bone anterior to frontal planes through the illustrated sections. This includes those moments acting on the contralateral hemimandible, whether behind or in front of the section plane. Symphysis frontal: moments about frontal planes through the symphyseal region summed anterior to the illustrated sections. Symphysis sagittal: moments about sagittal planes through the symphyseal region summed to the right of the illustrated sections. SI, superior-inferior; AP, anterior-posterior; ML, mediolateral.

Moments acting on the mandible about the (*X*), (*Y*) and (*Z*) axes throughout the gape cycle are shown in figure 1 for the no-screws model and in electronic supplementary material, figure S2 for the screws model. Across all three food types (nuts, grapes, dried fruit), the moments peak around 40% of the gape cycle (no-screws model: nuts, 40%; grapes, 35%; dried fruit, 39%; screws model: nuts 40%; grapes 37%, dried fruit 39%). In most cases peak moments are the highest during nut chewing and lowest during dried fruit chewing.

Across all foods, peak moments are experienced at similar locations in the mandible. Sagittal bending moments (about ML axes) are highest at the anterior ramus and posterior corpus of the balancing-side mandible (section 6), followed by the working-side anterior corpus below the bite point (left P4 and M1) (section 12), and the working-side anterior ramus (figure 1; electronic supplementary material, figure S2) (sections 5 and 6). Twisting moments about AP axes are highest on the working side, at the coronal section immediately behind the bite point (between working-side M1 and M2, section 11), and next highest at a sagittal section through the working-side (left) first incisor (symphyseal region, sections 6 and 7). Transverse bending moments (about SI axes) peak at symphyseal region sections 4–6, and are slightly lower at a coronal section through the P4s and back of the symphysis (section 13 on balancing side, section 12 on working side).

Although chews on all three food types show peak moments around the same time, there are differences between foods in the dynamics of these moments through time (in figure 1 and the electronic supplementary material, figure S2 the magnitudes of the moments are colour coded by the percentage of time through the gape cycle.) During nut and dried fruit chews, balancing-side sagittal bending moments (bottom left panel for each food type) show slow increases in moments up to *ca* 20% of the gape cycle. These moments then increase more rapidly during nut chewing than during dried fruit chewing: consequently, differences between nut and dried fruit chewing appear late in the power stroke. By contrast, during grape chewing these moments increase rapidly early in the gape cycle (10–15%), but more slowly later in the gape cycle, *ca* 20–40% of the gape cycle.

#### (ii) Deformation regimes

Chewing on all three foods is associated with three common deformation regimes: twisting of the rami, characterized by inversion of the coronoid process and eversion of the angle; sagittal bending; and lateral transverse bending (wishboning). During nut chewing, ramus twisting occurs bilaterally first, prior to 25% of the gape cycle, after which sagittal bending deformation and lateral transverse bending occur simultaneously, peaking at 40% of the gape cycle (electronic supplementary material, movie S1). Dried fruit chewing is accompanied by similar deformation regimes, although sagittal and transverse bending start slightly earlier (frame 18) and are lower in magnitude (electronic supplementary material, movie S2). During grape chewing, lateral transverse bending occurs earlier than the other two foods (frame 5) (electronic supplementary material, movie S3), corresponding to the early recruitment of the balancing-side superficial masseter and medial pterygoid (electronic supplementary material, figure S1), and the rapid raise in transverse bending moments (figure 1; electronic supplementary material, figure S2). Twisting of the rami begins around 12% of the gape cycle, being largest in magnitude on the working side. After 40% of the gape cycle, the balancing-side corpus and ramus undergo medial transverse bending until the jaw returns to its initial, undeformed state.

### (b) Dynamic changes in principal bone strains across food types

**Figure 2.**
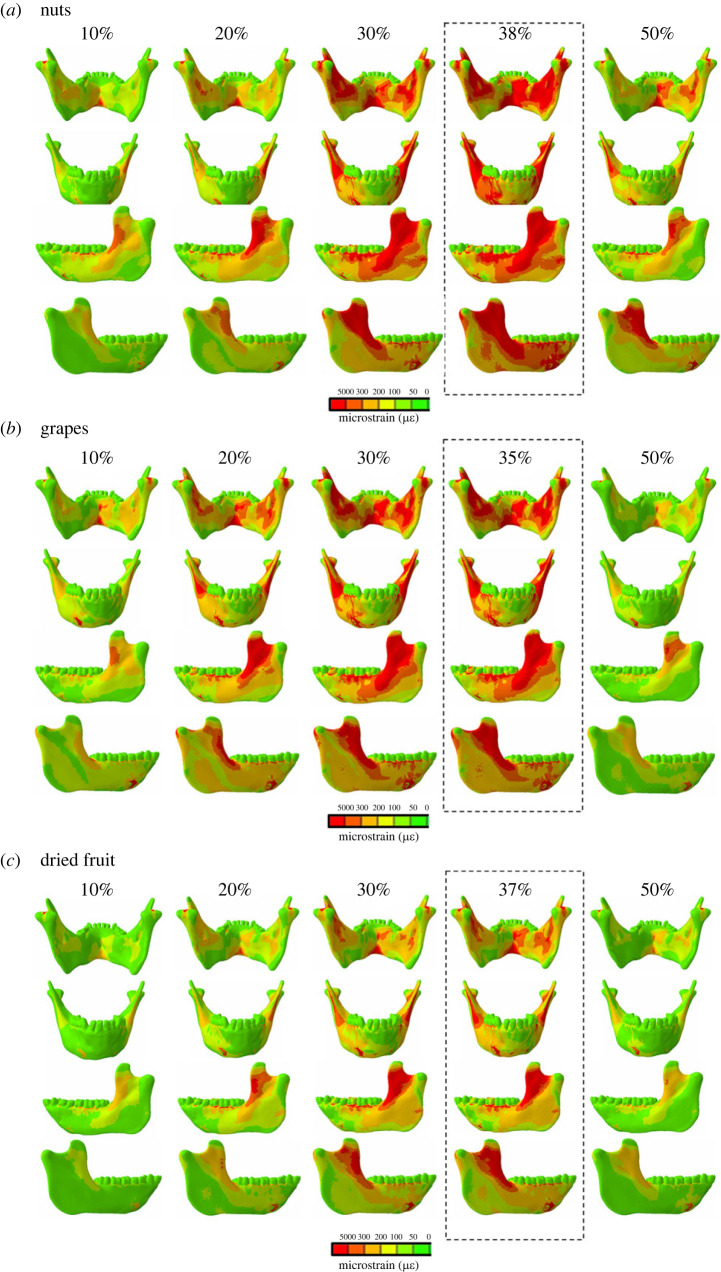
Maps of maximum principal strains (*ε*1) in the no-screws model surface at 10–50% of the gape cycle when the animal was chewing on (*a*) nuts, (*b*) grapes, and (*c*) dried fruit. Warmer and cooler colours represent higher and lower *ε*1 concentrations, respectively.

Peak principal strain magnitudes for all three food types were recorded at approximately 40% of the gape cycle when the highest moments were also recorded (no-screws model: grapes, 35%; dried fruit, 37%; nuts 38% (figure 2; electronic supplementary material, figure S3); screws model: grapes, 37%; dried fruit, 39%; nuts 40% (electronic supplementary material, figures S4–S5). Peak strains occur earlier in grape chews than in nut or dried fruit chews (figure 2; electronic supplementary material, figure S4). *ε*1 magnitudes are highest: bilaterally in the coronoid process and endocoronoid ridge, associated with twisting of the ramus; along the anterior border of the ramus, lateral and medial plana triangulares, external oblique lines, extramolar sulci and lateral prominences, associated with sagittal bending; and in the inferior transverse torus, medial prominence, torus triangularis and endocondylar ridge, associated with transverse bending. High strains were also recorded in the balancing-side superior transverse torus and planum alveolare, and in the working-side inferior transverse torus, associated with lateral transverse bending and twisting of the symphyseal region about a transverse axis (electronic supplementary material, movies S4–S6).

While strains late in the gape cycle (at and after 30% of the cycle) are the highest during nut chewing, followed by grapes, then dried fruit, strains early in the chewing cycle (10–30%) are the highest during grape chewing, followed by nuts, then dried fruit (figure 2; electronic supplementary material, figure S4). This strain differential between nuts and grapes early in the power stroke is reflective of the difference in EMG amplitudes, when EMG from the anterior and posterior temporales and the deep masseter are higher during grape than nut chewing (electronic supplementary material, figure S6).

#### (i) Balancing-side mandible

**Figure 3.**
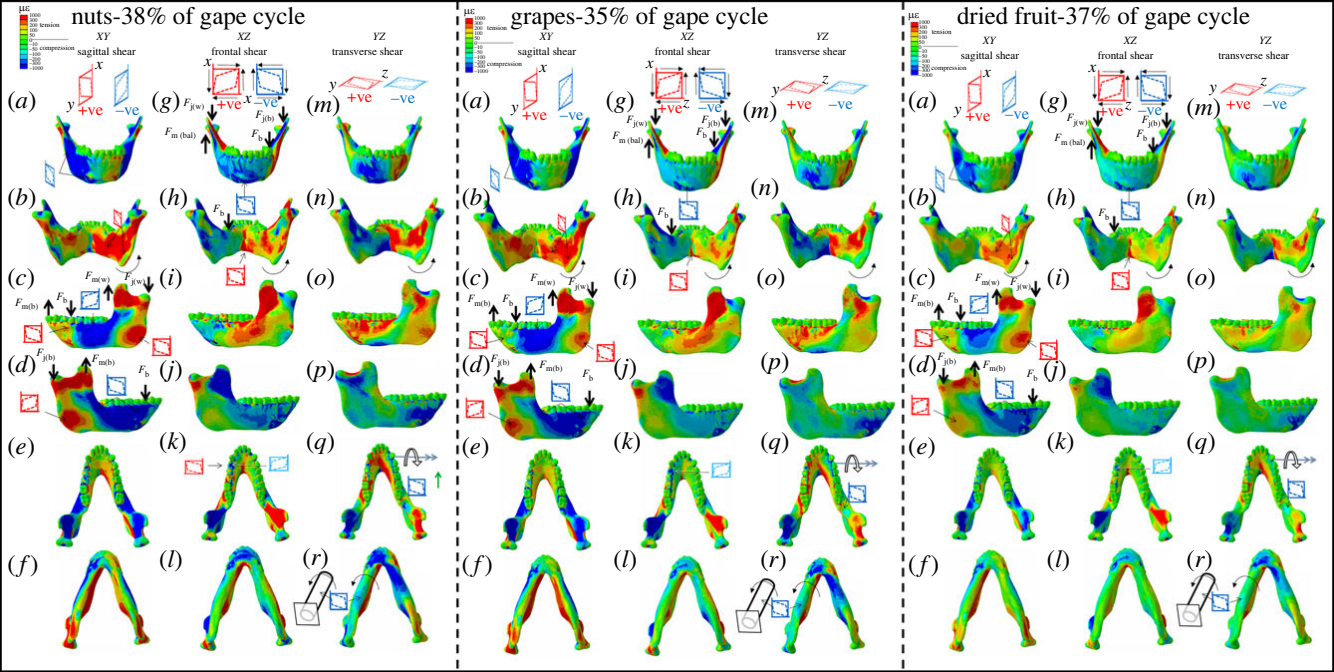
Shear strain regimes at approximately 40% of the gape cycle in the no-screws FEMs during simulated nut (38% of gape cycle), dried fruit (37%), and grape chewing (35%). (*a–f*) *XY* (sagittal) shear strains. (*g–l*) *XZ* (coronal). (*m–r*) *YZ* (transverse) planes. Scale is in microstrain, warm colours indicate positive strain (increase in relative length), cool colours indicate compressive strain (decrease in relative length).

Large positive ML (negative sagittal bending) moments (figure 1; electronic supplementary material, figure S2) acting on the balancing-side mandible are associated with high magnitudes of *ε*1 (figure 2; electronic supplementary material, figure S4) and tensile (positive) anteroposterior (*YY*) (electronic supplementary material, figures S7N & S8N) strains on the endocondylar ridge, recessus mandibulae, extramolar sulcus, alveolar prominence and planum alveolare. Tensile AP strains (electronic supplementary material, figures S7N & S8N) and increased transverse shear strains (figure 3*n*; electronic supplementary material, figure S9N) at the lingual corpus and ramus combined with compressive AP strains at the buccal corpus and ramus (figure 3*p*; electronic supplementary material, figure S9P) are associated with lateral transverse bending at the balancing-side angle, condyle, ramus and corpus.

The balancing-side mandible experiences low AP moments, which are negative at the posterior ramus and anterior corpus, positive at the posterior and mid corpus and peak at the level of the P4 (figure 1). This transition of the AP moments is reflected in the shear strains, which are positive at the posterior basal ramus (figure 3*r*; electronic supplementary material, figure S9R), planum alveolare and alveolar prominence (figure 3*q*; electronic supplementary material, figure S9Q), and negative at the basal corpus (figure 3*r*; electronic supplementary material, figure S9R) and extramolar sulcus (figure 3*q*; electronic supplementary material, figure S9Q). Negative vertical moments at the anterior ramus and corpus (figure 1; electronic supplementary material, figure S2) are associated with negative sagittal shear strain in the buccal corpus and external oblique line (figure 3*d*; electronic supplementary material, figure S9D).

#### (ii) Working-side mandible

The working-side mandible experiences lateral transverse bending, positive sagittal bending (negative ML moments) and twisting (negative AP moments) at the corpus. Moments peak at the P4 and M1 and decrease to almost zero behind the M3. At the ramus the dominant loading regime is negative sagittal bending with positive ML moments peaking at the level of the coronoid process (figure 1; electronic supplementary material, figure S2). Positive sagittal bending is associated with compressive AP strains in the buccal corpus (electronic supplementary material, figures S7O & S8O) and the planum alveolare near the bite point (electronic supplementary material, figures S7Q & S8Q) and tensile AP strains along the lingual corpus (electronic supplementary material, figures S7N & S8N), in particular in the basal corpus below the bite point (electronic supplementary material, figures S7R & S8R). Sagittal shear strains are positive at the buccal anterior corpus below the bite point, at the posterior-inferior ramus and the coronoid process, and negative behind the bite point and at the retromolar space (figure 3*c–f*; electronic supplementary material, figure S9C–S9F). Negative AP moments at the corpus (figure 1; electronic supplementary material, figure S2) are associated with positive and negative transverse shear strains respectively at the corpus (figure 3*o*; electronic supplementary material, figure S9O) and the base of the working-side mandible (figure 3*r*; electronic supplementary material, figure S9R).

#### (iii) Symphysis

Moments about the frontal and sagittal planes have similar patterns among food categories, but are highest at 40% of the gape cycle during nut chewing. In all food categories moments acting anterior to frontal planes through the symphysis are close to zero (as no forces are acting directly on the symphysis anterior to these planes). By contrast, moments acting to one side of sagittal planes through the symphysis can be quite large: transverse bending and axial twisting moments acting on the symphysis are larger than those acting on the balancing-side mandible. These moments are associated with the high magnitudes of tensile (*ε*1) (figure 2; electronic supplementary material, figure S4) and positive transverse shear (figure 3*n*; electronic supplementary material, figure S9N) strains in the balancing-side planum alveolare and with increased compressive (*ε*3) (electronic supplementary material, figures S3 & S5) and negative transverse shear strains (figure 3*n*; electronic supplementary material, figure S9N) in the working-side labial symphysis. The labial symphysis experiences increased sagittal shear moments towards the balancing side and more positive on the working side (figure 3*a*; electronic supplementary material, figure S9N). Frontal shear (figure 3*g*; electronic supplementary material, figure S9G) and transverse shear strains become more negative at the inferior aspect of the balancing-side labial symphysis in all food categories (figure 3*g*; electronic supplementary material, figure S9G), in particular during nut chewing.

## 4. Discussion

### (a) Overview

This is, to our knowledge, the first dynamic finite element simulation of mandible biomechanics published to date based on EMG and 3D jaw kinematic data, and validated against bone strain data collected *in vivo* during chewing on three different food categories: grapes, dried fruit, and nuts. The goal was to identify diet-related variation in loading regimes and in the times during the gape cycle when the mandible experiences peak strains, and to assess whether dynamic FEMs better reflect the biomechanical behaviour of the jaw during feeding than static FEMs. We showed that static FEMs do not fully capture the mechanics of the jaw during chewing, and therefore may not be the best tool for studies of the mechanical behaviour of the feeding system, and therefore to glean insights into the role of diet in morphological diversification and adaptive specialization of the mandible. Dynamic FEMs effectively represent the mechanical behaviour of the jaw during feeding by reflecting the asymmetry in jaw-adductor muscles activations during a gape cycle on loading, deformation and strain regimes [38,39].

### (b) What do static FEMs tell US about feeding system design?

The static FEMs previously published by our group on the same animal [5] show that the mandible is loaded and strained the most during nut chewing. Specifically, shelled nut chewing results in the highest transverse bending, anteroposterior and sagittal moments, followed by grapes and dried fruit. Static FE modelling also showed that the largest food-related variations in peak strain occurred in a strip from the lingual symphysis to the balancing-side corpus–ramus junction and along the balancing-side medial prominence and endocondylar ridge [5] suggesting that food effects on bone strain regimes are more salient in areas not traditionally investigated. However, a limitation of static FEMs is that any conclusions about the relationships between feeding behaviour, diet and jaw mechanics are based on a single time point in the gape cycle, thus failing to capture high bone strains that may occur at other times during the cycle.

### (c) Why are dynamic FEMs superior to static 3D FEMs in capturing the mechanical behaviour of the jaw during feeding on different foods?

Our dynamic FEMs show that peak strains and loading regimes vary not only across food categories and the gape cycle, but also extend beyond the anatomical areas reported by our static FEMs (figure 2). In particular, the dynamic FEMs captured peak strains across a larger area of the balancing-side corpus and ramus than previously reported, and across the coronoid process, the endocoronoid ridge, external oblique lines, extramolar sulci and lateral prominences and plana triangulares of the working-side hemimandible. This finding suggests that changes in cortical thickness in all these areas may reflect diet-related changes in bone strains.

Dynamic variation in moments across the three foods is reflected in variation in the deformation regimes of the hemimandibles at different degrees during the gape cycle (electronic supplementary material, movies S1–3). Nut chewing is associated with bilateral twisting of the rami early in the gape cycle, followed by simultaneous sagittal bending and lateral transverse bending, which peak at approximately 40% of the gape cycle, when bone strains and moments also peak (electronic supplementary material, movie S1). By contrast to nut chewing, during dried fruit and grape chewing the mandible experiences sagittal and transverse bending earlier in the gape cycle and at a lower magnitude (electronic supplementary material, movies S2 & S3). Lateral transverse bending early in the gape cycle during grape chewing corresponds with the early recruitment of the balancing-side superficial masseter and medial pterygoid and affects the strain regimes, particularly in the working-side corpus.

Our dynamic FEMs also confirmed that peak strain magnitude across all food categories occurred at approximately 40% of the gape cycle, when the anterior temporales, medial pterygoids and superficial masseters have started to decline, the balancing-side deep masseters and posterior temporales forces are about to peak, and force generated by the working-side deep masseters has declined (electronic supplementary material, figure S1). However, earlier in the gape cycle, peak bone strains were recorded during grape chewing associated with variations in the EMG activity of the temporales, and deep masseters (electronic supplementary material, figure S6).

As a result, future studies aimed to look at morphological measures of resistance associated with food related variations in bone strain should refrain from looking at one time point of the chewing cycle since that point may not be representative of the mechanical behaviour of the mandible during a complete gape cycle.

### (d) Study implications

While our study was conducted on a rhesus macaque, similarities in feeding mechanics and bone strains between macaques [5,6,38,46], chimpanzees [8] and humans [9,34,47] make our findings and methods relevant to broader anthropological and oral and maxillofacial research. Most previous comparative, anthropological studies of the relationship between diet and mandible morphology have focused on the cross-sectional morphology of the symphysis and corpus, or on the shape of the mandibular condyle [46,48–52]. As we have argued elsewhere, these studies have not revealed strong dietary signals in the mandible [39]. The results of our dynamic FEM analysis suggest that study of the morphology of other regions of the mandible might also be of interest.

Our dynamic FEM also suggests that clinical studies of mandible function would also benefit from dynamic analyses. Most biomechanical analyses of human mandibles discuss single loading regimes, such as biting on the incisors, or biting on the molars [34,53,54]. To date no dynamic FEMs of the human mandible during a complete chewing sequence have been published. These dynamic models will be necessary in order to fully compare the efficacy of, e.g. different methods of fixing mandible fractures, replacing TMJs, or distraction osteogenesis.

### (e) Study limitations

(i) The model used in our previously published static FEMs had screws implanted in the anterior mandible for the measurement of mandible three-dimensional kinematics. These screws had activated bone remodelling the impact of which on jaw mechanics was unknown. To ensure that the screws and the associated bone remodelling do not compromise model performance, we conducted this current dynamic study using both a model with and without the screws and calluses. Our results show that the screws and the calluses minimally impact deformation, loading and strain regimes at the lingual symphysis (electronic supplementary material, movie S7), showing that experimental interventions aimed at collecting high resolution three-dimensional kinematic mandible data do not impact the mechanical function of the jaw during feeding. However, we note that, while the ‘no screws’ model is closer to biological reality than the ‘screws’ model, it still shows some artefacts i.e. isolated high strained elements in the labial symphysis. This is because the radiodensity of the CT scans around the anterior mandible was impacted by the calluses, so that during the material assignment process some isolated elements at the labial symphysis have low Young's moduli values and high strains.
(ii) During removal of screws and calluses, we had to assign the PDL to the cortical bone. However, although the PDL was not modelled as a separate tissue, it was still accounted for in the simulation considering that the material properties assignment of the cortical bone was conducted using our previously published theoretical model [6,40]. Under this model, the electron densities of the cortical bone were converted to physical densities using a piecewise linear model that relates electron and physical densities in the CT scan phantom data. The linear model then related the bone density to Young's moduli and Poisson's ratios. Thus, although the PDL is not modelled as a separate tissue, it has been taken into consideration.
(iii) Myological values (forces and activation patterns) were collected *in vivo* on an animal with screws implanted on the anterior symphysis to measure jaw kinematics. The screw implantation occurred months before the experiments and at the time of the experiment the animal showed no evidence of pain or discomfort. Moreover, Ross *et al*. [55] showed that even strain gage placement had no effect on jaw kinematics in capuchin monkeys. In any event, any potential impact on muscle activation and force production owing to experimental intervention would be consistent across all models and does not impact model comparisons for the scope of this study.

## 5. Conclusion

Three-dimensional FEMs that are validated against bone strain data and built using *in vivo* three-dimensional jaw joint kinematics and muscle EMG data are important tools for reconstructing form–function relationships of the masticatory system. Here we show that dynamic FEMs are more informative than static FEMs in capturing the mechanical behaviour of the masticatory system, revealing loading, strain and deformation regimes that the static FEMs did not capture. Static FEMs are inevitably used in the field of feeding biomechanics when *in vivo* data on feeding behaviour are not available. However, we urge researchers to acknowledge the limitations of using static FEMs when drawing conclusions about relationships between feeding behaviour and diet. We also outline the need for more muscle EMG and strain gauge data to reconstruct validated and subject specific dynamic FEMs to better understand how fundamental constraints in the feeding system design affect feeding performance and diet.

## Data Availability

The datasets supporting this article have been uploaded as part of the electronic supplementary material [56]. All FEM information are available from Figshare: https://figshare.com/s/d35b7705d5cf86608f7a.

## Endnotes

1 We recognize that gummy bears are not dried fruits. However, experimental studies [39] found that dried gummy bears have a fragmentation index (ExR)^0.5^ comparable to dried apricot and raisin, so we grouped gummy bears with dried fruits.
2 Macaque jaw-adductor muscles express varying amounts of slow (major histocompatibility complex (MHC)-1) and fast (MHC-M) myosin heavy chain isoforms [42]. Isometric tension values (*P*_o_) from single-fibre studies report *P*_o_ of 22.5 N cm^−2^ and 38 N cm^−2^ for MHC-1 and MHC-M, respectively [43]. Our *P*_o_ of 30 N cm^−2^ represents the average of these two *P*_o_ estimates.
